# Anion-Sensitive Regions of L-Type CaV1.2 Calcium Channels Expressed in HEK293 Cells

**DOI:** 10.1371/journal.pone.0008602

**Published:** 2010-01-06

**Authors:** Norbert Babai, Nataly Kanevsky, Nathan Dascal, George J. Rozanski, Dhirendra P. Singh, Nigar Fatma, Wallace B. Thoreson

**Affiliations:** 1 Department of Ophthalmology & Visual Science, University of Nebraska Medical Center, Omaha, Nebraska, United States of America; 2 Department of Physiology and Pharmacology, Sackler School of Medicine, Tel Aviv University, Ramat Aviv, Israel; 3 Department of Cellular & Integrative Physiology, University of Nebraska Medical Center, Omaha, Nebraska, United States of America; Oregon Health & Science University, United States of America

## Abstract

L-type calcium currents (I_Ca_) are influenced by changes in extracellular chloride, but sites of anion effects have not been identified. Our experiments showed that CaV1.2 currents expressed in HEK293 cells are strongly inhibited by replacing extracellular chloride with gluconate or perchlorate. Variance-mean analysis of I_Ca_ and cell-attached patch single channel recordings indicate that gluconate-induced inhibition is due to intracellular anion effects on Ca^2+^ channel open probability, not conductance. Inhibition of CaV1.2 currents produced by replacing chloride with gluconate was reduced from ∼75%–80% to ∼50% by omitting β subunits but unaffected by omitting α_2_δ subunits. Similarly, gluconate inhibition was reduced to ∼50% by deleting an α1 subunit N-terminal region of 15 residues critical for β subunit interactions regulating open probability. Omitting β subunits with this mutant α1 subunit did not further diminish inhibition. Gluconate inhibition was unchanged with expression of different β subunits. Truncating the C terminus at AA1665 reduced gluconate inhibition from ∼75%–80% to ∼50% whereas truncating it at AA1700 had no effect. Neutralizing arginines at AA1696 and 1697 by replacement with glutamines reduced gluconate inhibition to ∼60% indicating these residues are particularly important for anion effects. Expressing CaV1.2 channels that lacked both N and C termini reduced gluconate inhibition to ∼25% consistent with additive interactions between the two tail regions. Our results suggest that modest changes in intracellular anion concentration can produce significant effects on CaV1.2 currents mediated by changes in channel open probability involving β subunit interactions with the N terminus and a short C terminal region.

## Introduction

L-type Ca^2+^ channels are involved in many vital functions including contraction of skeletal, smooth, and cardiac muscle; release of neurohormones and neurotransmitters; and gene expression [1]–[4]. They can be regulated by many different mechanisms [1], [5]–[7]. One poorly understood mechanism involves the effects of anions on I_Ca_. Replacing Cl^−^ with various substituting anions influences many Ca^2+^-mediated processes including contractility of cardiac and skeletal muscle, hormone secretion, and neurotransmitter release [8]–[16]. An important contributor to these anion effects is the modulation of L-type Ca^2+^ currents (I_Ca_) [8]–[10], [17], [18]. Large reductions in extracellular chloride produced by replacing Cl^−^ with gluconate or perchlorate can substantially inhibit I_Ca_ [8], [18]. Replacing a small amount of Cl^−^ with gluconate can also produce significant inhibitory effects [8], [18] but small concentrations of perchlorate can have stimulatory effects [13], [14], [16]. The inhibition of I_Ca_ produced by replacing chloride with gluconate and the enhancement of I_Ca_ caused by low concentrations of perchlorate have both been shown to be due to the actions of anions at intracellular sites which alter the open probability of Ca^2+^ channels [16], [17].

Chloride and other anions influence the structure and activity of many different proteins including opsins [19], intracellular Ca^2+^ channels [20], hemoglobin [21], [22], albumin [23], PDZ domains [24], K^+^ channels [25], [26], kainate receptors [27], serine/threonine kinases [28]–[30], and G proteins [31]. Anion effects on protein function typically involve binding to positively charged lysine or arginine residues. In the present study, we expressed different subunit combinations and CaV1.2 mutant channels in HEK293 cells to analyze channel regions responsible for the anion sensitivity of L-type Ca^2+^ channels. We identified two anion-sensitive regions of L-type Ca^2+^ channels: 1) a short region of the C terminus in which a pair of neighboring arginine residues is particularly important and 2) interactions between accessory β subunits and a short region of the N terminus. Consistent with previous reports, we found that anions act inside the cell to modulate Ca^2+^ channel open probability and low anion concentrations can produce significant effects on I_Ca_. These results suggest that modest, physiologically-attainable changes in the intracellular levels of chloride or other anions can influence the activity of L-type Ca^2+^ channels by actions at multiple channel regions and thus potentially influence Ca^2+^-dependent processes in many different tissues throughout the body.

## Materials and Methods

### Ethics Statement

All animal procedures were approved by University of Nebraska Medical Center Institutional Animal Care and Use Committee, and conducted according to the *Guide for the Care and Use of Laboratory Animals*, published by the National Institutes of Health (NIH Publication No. 85-23, Revised 1996).

HEK293 cells were grown in DMEM supplemented with 10% fetal bovine serum (FBS) and 50 U/ml gentamicin and maintained at 37°C in a humidified incubator with 5% CO_2_. Upon nearing confluence, cells were dissociated enzymatically with trypsin-EDTA and plated overnight on 13mm diameter plastic cover-slips (NUNC, Rochester, NY USA) in 35×10mm tissue culture dish (Falcon, Franklin Lakes, NJ. USA). Cover-slips were transferred to 24 well tissue culture plates with 0.5 ml grown media without antibiotic and FBS (Falcon, Franklin Lakes, NJ. USA) and transiently transfected using Lipofectamine 2000 (Invitrogene, Carlsbad, CA). cDNA from the α1 subunit (1 µg) was cotransfected with one of the rat β subunits (1 µg) (i.e., β_2a_, β_1b_, β_3_, or β_4_) and the rat α_2_δ (1 µg) subunit. The α1 subunits used in this study were a short N-terminal isoform of CaV1.2 derived from rat brain [32] (M67515), a long N-terminal isoform of rabbit CaV1.2 derived from cardiac tissue [33] (X15539), and various mutants of rabbit CaV1.2. The Δ139/Δ1665 double mutant was constructed by cutting and ligating appropriate parts of the Δ139 and Δ1665 mutants. Details of the other CaV1.2 mutants are described elsewhere [33]–[36]. We co-transfected cells with enhanced green fluorescent protein (eGFP) (1 µg; Clontech, Cambridge, UK) as a marker plasmid. Transfected cells were incubated in transfection medium for 6–8 hrs. before replacing it with standard growth medium. Cells were used for recording 24–72 hrs. after transfection.

Cells were superfused at room temperature using a single-pass, gravity-fed perfusion system (1 ml/min) with an oxygenated medium containing (in mM): 130 NaCl, 5 KCl, 5 BaCl_2_, 10 4-*N*-2-hydroxyethylpiperazine-*N*′ 2-ethanesulfonic acid (HEPES), 10 glucose (pH 7.4). For anion replacement experiments, we replaced NaCl and KCl but not BaCl_2_. Gluconate weakly chelates Ba^2+^ and calculations using WCabuf (G. Droogmans, Leuven, Belgium) indicate that the free Ba^2+^ concentration is reduced by gluconate replacement from 5 mM to 4.34 mM. However, in control experiments, we found that reducing Ba^2+^ to 4.34 mM did not significantly reduce CaV1.2 currents (−2.7±2.2%, N = 7, P = 0.996).

Whole cell recordings were obtained using patch electrodes pulled from borosilicate pipettes (1.2 mm outer diameter, 0.95 mm inner diameter, with internal filament) using a Narishige PP-830 vertical puller. The recording pipettes had tips of 1–2 µm outer diameter (R = 7–10 MΩ) and were filled with a solution containing (in mM): 125 CsCl, 10 tetraethyl ammonium chloride (TEACl), 10 HEPES, 3 ethylene glycol *bis* (β-aminoethyl ether) N, N, N‵, N‵-tetraacetic acid (EGTA), 1 ATP, 0.5 GTP, 3 MgCl_2_, 1 CaCl_2_ (pH 7.2). The low Cl^−^ pipette solution contained (in mM): 125 Cs gluconate, 10 TEACl, 10 HEPES, 3 EGTA, 1 ATP, 0.5 GTP, 3 MgCl_2_, 1 CaCl_2_ (pH 7.2). The reference electrode was connected to the bath by a 3 M KCl/agar bridge. With the agar bridge in place, the liquid junction potential changed by ≤1 mV when chloride in the bathing medium was replaced with gluconate.

HEK cells were voltage clamped at −70 mV using an Axopatch 200B or Multiclamp amplifier (Axon Instruments, Foster City, CA). The barium current (I_Ba_) was typically recorded with a ramp voltage protocol (−90 to +60 mV, 0.5 mV/ms). I_Ba_ was fit with a Boltzmann function adjusted for driving force. The fitting region extended from baseline to 10 mV beyond the peak inward current. Current/voltage relationships measured with ramp protocols matched current/voltage relationships determined from steady state currents evoked by depolarizing steps (100 ms, 10 mV increments; Fig. S1). Currents were acquired using PClamp 9.2 with a Digidata 1322 interface (Axon Instruments). Currents were leak-subtracted *post-hoc* or by using P/8 protocols.

Single ventricular myocytes were dissociated from isolated, perfused rat hearts by a collagenase digestion procedure described previously [37]. Dissociated myocytes were suspended in DMEM and stored in an incubator at 37°C. Aliquots of myocytes to be studied were transferred to a cell chamber mounted on the stage of an inverted microscope and superfused with an external solution containing (in mM): 138 NaCl; 4 CsCl; 0.5 MgCl_2_; 1.8 CaCl_2_; 10 glucose; 5 HEPES (pH 7.4).

For measurements of I_Ca_ from ventricular myocytes, currents were evoked by 300 ms depolarizing pulses to test potentials between −40 and +60 mV (0.2 Hz). The holding potential in all experiments was −80 mV and 100 ms prepulse to −50 mV was applied to inactivate the fast Na^+^ current. At each test potential the amplitude of I_Ca_ was measured as the difference between the peak inward current and the current level at the end of the depolarizing clamp pulse. Data were normalized as current densities by dividing measured current amplitude by whole-cell membrane capacitance (pA/pF).

For mean/variance analysis of single channel current amplitudes from CaV1.2 channels expressed in HEK293 cells, we applied 100 test pulses (5 ms) from −70 to +50 mV. For these experiments, currents were filtered at 5 kHz and access resistance was compensated 80–90%. For P/200 subtraction of passive and capacitative currents, we summed two trials involving 100 tests pulses of 1.2 mV amplitude recorded immediately before and after the test pulse series. The mean amplitude and variance was determined at each time point during the tail current. The mean/variance relationship was fit with a parabolic function:
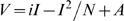
where I = mean whole cell current amplitude, i = single channel current amplitude, N = channel number, A = offset, and V = variance.

Cell-attached patch recordings of single CaV1.2 channels were obtained using pipettes coated with Sylgard (Dow Corning, Midland, MI) and filled with 82 mM BaCl_2_. Recordings were low pass filtered with a cutoff frequency of 2 kHz and digitized at 50 µs/sample. In the cell-attached patch configuration, the transmembrane voltage across the patch is a sum of the cell membrane potential and voltage applied by the amplifier. Using gramicidin (5 µg/ml) as a perforating agent along with a pipette solution containing (in mM): 98 KCH_3_SO_4_; 44 KCl; 3 NaCl; 5 HEPES; 3 EGTA; 3 MgCl_2_; 1 CaCl_2_; 2 glucose; 1 Mg-ATP; 1 GTP (pH 7.2), we found that the resting membrane potential of HEK293 cells averaged −51.6±1.0 mV (N = 10). Patches were held at +10 mV yielding a net trans-membrane voltage of −61.6 mV across the membrane patch. Channel openings were stimulated with 5 s test pulses to depolarize the membrane patch by 50 mV to −11.6 mV. Gluconate replacement depolarized HEK293 cells by ∼10 mV to −39.1±1.5 mV (N = 10). To compensate for the depolarization produced by gluconate, we analyzed test steps that depolarized the patch by only 40 mV rather than 50 mV. Single channel amplitude and open probability were analyzed during 5 s test pulses using Clampfit software (Axon Instruments).

Unless otherwise specified, all chemicals were obtained from Sigma Chemicals (St. Louis, MO). The criterion for statistical significance was chosen to be P<0.05 and evaluated with Student's T-test or ANOVA using GraphPad Prism 4. Variability is reported as ±SEM.

## Results

### Effects of Chloride Replacement on CaV1.2 Currents

α_1_ subunits of CaV1.2 channels were co-expressed with EGFP, β_2a_ and α_2_δ subunits in HEK293 cells. Currents were measured with a ramp voltage protocol and 5 mM Ba^2+^ was used as a charge carrier to enhance currents through Ca^2+^ channels. Fitting ramp-evoked I_Ba_ with a Boltzmann function adjusted for driving force yielded a midpoint activation (V_50_) of −10.5±0.9 mV and slope factor of −8.3±0.4 (N = 17; Table 1) consistent with previous reports [38]. Untransfected HEK293 cells exhibit a small endogenous I_Ba_ [39] which averaged 0.065±0.006 pA/pF (N = 33), much smaller than currents in CaV1.2-transfected cells (22.0±4.5 pA/pF, N = 17).

**Table 1:** Boltzmann parameters and current densities for different channel combinations.

	V_50_ (mV)	Slope factor	G_max_ (nS)	Current density (pA/pF)	N
**CaV1.2/α_2δ_/β_2A_**	−10.5±0.9	−8.3±0.4	3.2±0.5	22±4.5	17
**CaV1.2/α_2δ_/β_1B_**	−12.5±3.1	−9.5±2.0	1.8±0.3	21.6±6.0	6
**CaV1.2/α_2δ_/β_3_**	−10.6±3.1	−10.9±1.5	3.6±1.2	16.2±4.2	13
**CaV1.2/α_2δ_/β_4_**	−9.2±3.9	−11.3±0.8	1.9±0.2	20±5.8	5
**CaV1.2/α_2δ_**	−9.6±1.9	−10.7±0.8	2.8±1.2	8.5±1.8	14
**CaV1.2/β_2A_**	−4.6±1.1	−9.8±1.6	1.6±0.7	6.0±3.0	4
**CaV1.2**	−8.1±1.6	−11.0±0.5	1.9±0.5	7.5±1.7	6
**LongNT/α_2δ_/β_2A_**	−9.8±1.8	−11.4±0.8	2.1±0.5	18.1±5.6	13
**LongNT/α_2δ_**	−8.8±5.6	−13.8±1.1	2.0±0.7	4.4±1.0	6
**Δ139/α_2δ_/β_2A_**	−16.5±1.8	−10.0±0.8	3.2±0.7	12.5±2.1	9
**Δ6–20/α_2δ_/β_2A_**	−12.6±2.4	−11.0±0.8	2.4±0.7	11.6±1.6	6
**Δ6–20/α_2δ_**	−13.5±0.8	−7.3±1.3	1.5±0.4	2.5±0.5	7
**Δ1665/α_2δ_/β_2A_**	−6.5±1.8	−11.9±0.7	1.8±0.2	3.5±0.5	5
**Δ1701RRQQ/α_2δ_/β_2A_**	−11.6±1.7	−10.4±1.0	2.1±0.3	15.6±2.7	10
**Δ139/Δ1665/α_2δ_/β_2A_**	−6.8±3.0	−10.2±0.6	1.3±0.2	12.0±3.0	10
**Δ1665/α_2δ_**	−4.9±3.9	−10.2±1.6	1.1±0.3	3.7±1.5	5
**Δ139/Δ1700/α_2δ_/β_2A_**	−13.6±1.2	−9.6±0.6	2.3±0.5	9.5±2.7	8

**Figure 1:**
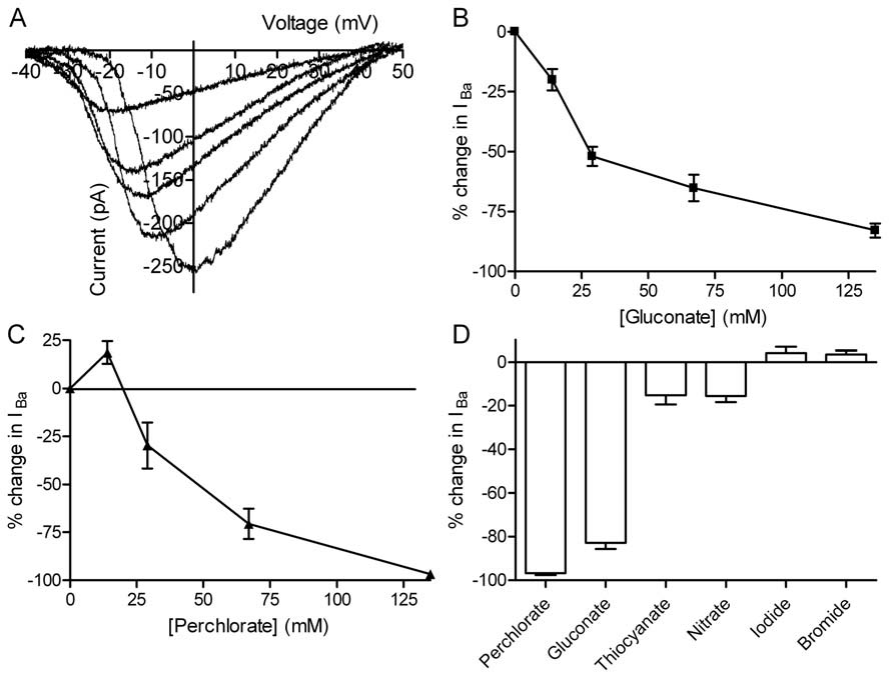
Effects of gluconate and other anions on CaV1.2 currents. A. CaV1.2 currents (short N-terminal isoform) were progressively inhibited by replacing increasing amounts of extracellular chloride with gluconate. 5 mM Ba^2+^ was used as a charge carrier and currents were evoked by a ramp voltage protocol (−90 to +60 mV, 0.5 mV/ms). Traces show I_Ba_ recorded in control conditions and after 2 min superfusion with solutions containing increasing concentrations of gluconate (14 mM, 28 mM, 68 mM, and 135 mM). Leak currents were removed by subtracting the ohmic conductance measured below I_Ba_ threshold. B. Increasing gluconate concentration caused a concentration-dependent increase in inhibition of the peak amplitude of CaV1.2 currents. A gluconate concentration of 14 mM caused 20.1±4.4% (N = 14) inhibition whereas 135 mM gluconate caused 82.9±2.9% inhibition (N = 28). C. Replacing 14 mM Cl^−^ with equimolar perchlorate increased CaV1.2 currents (18.7±5.8%, N = 6) but further increases in perchlorate concentration caused a concentration-dependent inhibition of CaV1.2 currents with −96.8±0.9% inhibition (N = 7) at a perchlorate concentration of 135 mM. D. Bar graph comparing effects of replacing 135 mM Cl^−^ with equimolar quantities of perchlorate (N = 7), gluconate (N = 28), thiocyanate (N = 8), nitrate (N = 9), iodide (N = 10) and bromide (N = 9). All of these experiments were performed using α_1_/β_2a_/α_2_δ.

As illustrated in Fig. 1, CaV1.2 currents were strongly inhibited by replacing extracellular chloride with equimolar gluconate. Increasing the concentration of gluconate caused progressive inhibition of I_Ba_, attaining 82.9±2.8% (N = 28) inhibition of peak amplitude at 135 mM gluconate. Similar strong inhibition by gluconate replacement was observed when CaV1.2 currents were measured using voltage step protocols (−76.9±3.7%, N = 8, Fig. S1). The small endogenous I_Ba_ was not significantly inhibited by gluconate replacement (−7.2±5.5%, N = 31, P = 0.57). Fig. 1B plots the average change in I_Ba_ amplitude observed with different concentrations of gluconate. Consistent with experiments on L-type currents *in vivo* [8], replacing only 14 mM chloride with gluconate significantly inhibited I_Ba_.

Inhibition by gluconate was accompanied by a negative voltage shift in I_Ba_ (Fig. 1A). At a gluconate concentration of 135 mM, V_50_ determined from the Boltzmann fit to the average I_Ba_ from 13 cells shifted by −8.1 mV. This negative shift in V_50_ is due to effects of gluconate on membrane surface charge [11], [16], [18], [40]. By promoting a leftward shift in the voltage-dependence of outward K^+^ or Cl^−^ currents [9], [11], [14], [16], [18], [41], [42], surface charge effects also contributed to a small negative shift in the net whole cell reversal potential in some cells. To test for the possibility that changes in driving force might have significantly influenced measurements of the inhibitory effects of gluconate on peak amplitude of I_Ba_, we analyzed effects of gluconate on the maximum conductance of I_Ba_ (G_max_) determined from the Boltzmann function fit to the average I_Ba_ waveform. Similar to gluconate's effects on I_Ba_ amplitude, G_max_ was reduced 72% by replacing 135 mM Cl^−^ with equimolar gluconate. Inhibition of I_Ba_ by gluconate can enhance the contribution of residual currents to the net whole cell current. The impact of residual K^+^ currents on measured changes in I_Ba_ peak amplitude appeared minimal since we observed a similar reduction in amplitude when we conducted gluconate replacement experiments using a higher concentration of TEA (60 mM; −74.7±3.4%, N = 9, P = 0.14, unpaired t-test).

The identity of the substituting anion influenced the effects of chloride replacement. Perchlorate is a low charge density anion that has been shown to alter I_Ba_ and Ca^2+^-mediated processes in many cell types [13], [14], [16], [18]. By contrast with inhibitory effects of 14 mM gluconate, 14 mM perchlorate enhanced CaV1.2 (Fig. 1C). This is consistent with the enhancement of L-type I_Ca_ produced by low concentrations of perchlorate in various tissues [13], [14], [16]. However, higher perchlorate concentrations caused a progressive inhibition of CaV1.2 currents with almost complete inhibition achieved at a concentration of 135 mM perchlorate (−96.8±0.9%, N = 7). To test whether this inhibitory effect was also observed with L-type Ca^2+^ channels *in vivo*, we recorded I_Ca_ from acutely isolated rat ventricular muscle cells and examined the effects of 135 mM perchlorate. Similar to the inhibition observed with expressed CaV1.2 channels, we found that 135 mM perchlorate inhibited the peak amplitude of I_Ca_ in ventricular muscle cells by 78.5±2.0% (N = 6). The finding that low concentrations of perchlorate enhanced I_Ba_ whereas high concentrations inhibited I_Ba_ could be explained by the presence of different anion interaction sites that exhibit different affinities for perchlorate.

We tested effects of other anions to determine whether inhibition of CaV1.2 currents follows the Hofmeister series (perchlorate>iodide>nitrate>bromide∼chloride), as found with L-type I_Ca_ in photoreceptors [18], [40], [43]. Replacing 135 mM chloride with bromide (+3.5±1.8%, N = 9) produced no significant effect on CaV1.2 current amplitude (Fig. 1D). Iodide also had little effect on CaV1.2 currents (+4.1±2.8%, N = 10). Nitrate replacement caused a modest but significant inhibition (−15.5±2.8%, N = 9; P = 0.0006). Interestingly, thiocyanate, which occupies a similar position in the Hofmeister series as perchlorate, caused only modest inhibition of −15.2±4.3% (N = 8). However, this is consistent with findings that thiocyanate has little effect on I_Ca_ in ventricular muscle cells [10]. Inhibitory effects of anions on CaV1.2 thus differed somewhat from the Hofmeister sequence, following the order perchlorate>gluconate>>thiocyanate∼nitrate>iodide∼bromide∼chloride.

### Mechanisms of Anion Regulation

**Figure 2:**
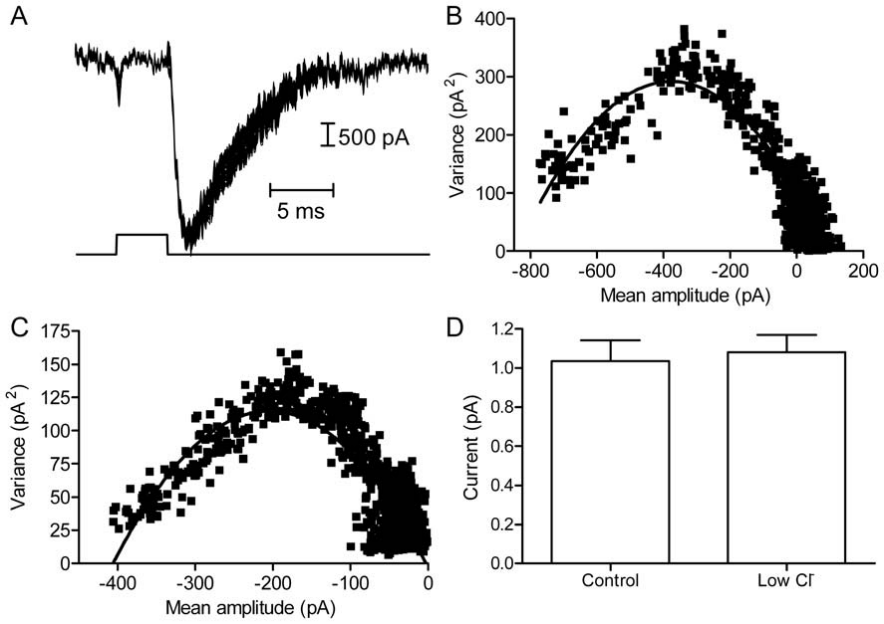
Replacing chloride with gluconate did not alter single channel amplitude of CaV1.2 currents. A. Overlaid traces showing a series of 100 test steps (5 ms, −70 to +50 mV). Passive membrane properties were subtracted using a P/200 protocol. B. The mean amplitude of the I_Ba_ tail current (A) was plotted against the variance at each time point (beginning at the peak inward current). The relationship between mean and inter-trial variance at different time points was fit with a parabolic function (see Methods). The best fit with this function indicates that I_Ba_ resulted from 746±21 channels with a single channel amplitude averaging −1.01±0.02 pA. C. Replacing 29 mM Cl^−^ with equimolar gluconate inhibited the amplitude of I_Ba_ by 48% in this cell, but the best fit parabola to the mean/variance relationship showed little change in single channel current amplitude (−1.15±0.03 pA). D. On average, lowering chloride had no significant effect on single channel current amplitude (control: −1.04±0.11 pA, N = 19; 29 mM gluconate: −1.08±0.09 pA, N = 19, P = 0.76).

Previous studies have shown that the effects of anions on L-type I_Ca_ in pancreatic beta cells and retinal photoreceptors are due to changes in Ca^2+^ channel open probability and not to changes in single channel conductance [16]–[18]. We tested whether gluconate influences single channel current amplitude of CaV1.2 currents by using mean/variance analysis techniques. To do so, we activated I_Ba_ using a series of 100 brief test steps from −70 to +50 mV. We measured the I_Ba_ tail current after P/200 subtraction of passive membrane properties (Fig. 2A). The relationship between mean and inter-trial variance at different time points was fit with a parabolic function. In the example shown in Fig. 2B, the best fit parabolic function to the mean/variance relationship (see Methods) indicated that the tail current resulted from 746±21 channels with a single channel current amplitude averaging −1.01±0.02 pA. Lowering extracellular chloride level from 143 to 116 mM by replacement with equimolar gluconate inhibited I_Ba_ by 48% in this cell (Fig. 2C), but had little effect on single channel current amplitude (−1.15±0.03 pA, Fig. 2C). In 19 cells, lowering chloride by 29 mM through gluconate replacement produced no significant effect on single channel current amplitude (Fig. 2D).

**Figure 3:**
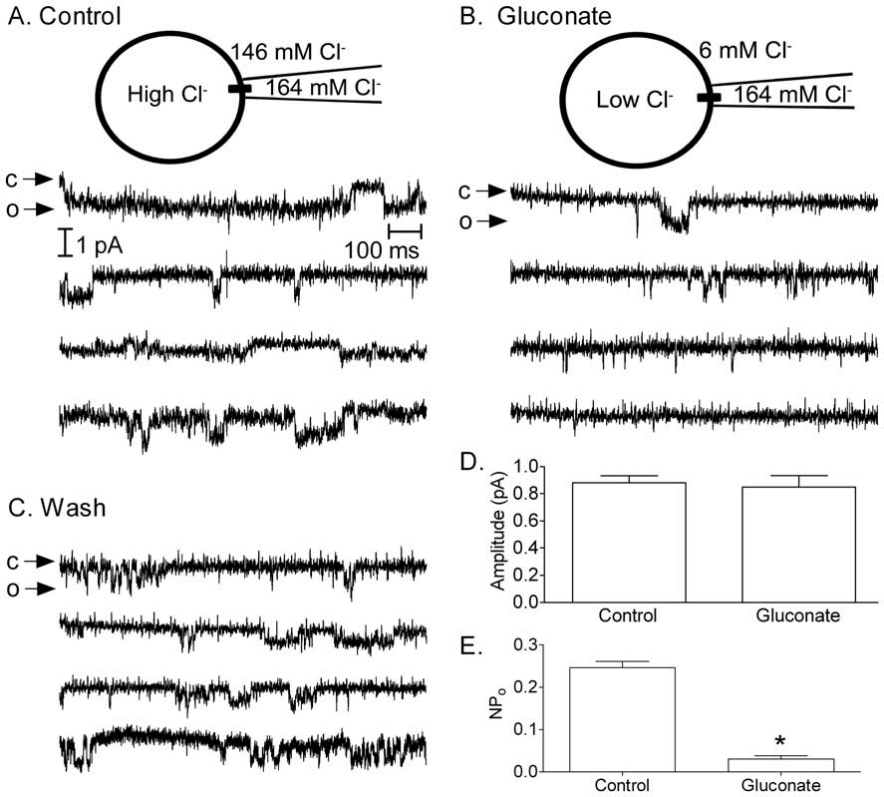
Gluconate replacement acts inside the cell to reduce Ca^2+^ channel open probability. CaV1.2 channel openings were recorded using the cell-attached patch configuration. Inward currents into the cell are shown as downward deflections. Panel A shows a sequence of 4 sweeps in control conditions. Panel B shows that that the number of channel openings dropped dramatically when extracellular Cl^−^ was replaced with gluconate (135 mM). Panel C shows that channel openings recovered after washout. The extracellular channel surface was continuously exposed to 164 mM Cl^−^ in the recording pipette suggesting that the reduction in channel opening are due to intracellular effects of gluconate replacement. For illustration, currents were smoothed by Butterworth filtering (8-pole, 800Hz). D. The amplitude of single channel currents was not significantly reduced by gluconate (N = 7, P = 0.38, paired t-test). C. Channel open probability (NP_o_) was significantly reduced by gluconate replacement (N = 7, P<0.0001, paired t-test). Control data were analyzed from sweeps obtained at an estimated trans-membrane potential across the patch of ∼−12 mV. Gluconate sweeps were analyzed using a 10 mV more positive test pulse to compensate for the ∼10 mV depolarization of HEK293 cells produced by the gluconate solution.

For a more direct test of the hypothesis that intracellular anion changes alter channel open probability, we obtained cell-attached patch recordings of single CaV1.2 channels. We employed an experimental approach used previously to study anion sensitivity of single L-type Ca^2+^ channels in photoreceptor terminals [17]. The recording pipette was filled with 82 mM BaCl_2_ to enhance the amplitude of single channel Ca^2+^ currents. Channel openings were stimulated by applying test steps (5 s) to depolarize the membrane patch to ∼−12 mV (see Methods). The absence of cations other than Ba^2+^ in the pipette and the high concentration of Cl^−^ favoring Cl^−^ influx (outward current) indicate that inward channel currents were carried by Ba^2+^. Consistent with previously reported values for L-type Ca^2+^ channels (e.g., [19], [44]–[50]), the peak mean open probability of channel openings during 5 s test pulses averaged 0.23±0.03 (N = 13) and the slope conductance determined from channel currents measured at three different test potentials averaged 19.8±3.9 pS (N = 6). As illustrated in Fig. 3, replacing Cl^−^ with gluconate in the bathing medium caused a substantial reduction in open probability from 0.25±0.01 to 0.03±0.01 (N = 7, P<0.0001, paired t-test). Because the extracellular membrane surface was exposed to 164 mM Cl^−^ in the recording pipette, the reduction in Ca^2+^ channel openings was presumably due to changes in intracellular anion levels resulting from bath application of the gluconate solution. Consistent with an efflux of Cl^−^ through endogenous Cl^−^ channels in HEK293 cells [41], the gluconate test solution depolarized HEK293 cells by ∼10 mV, from −51.6±1.0 mV to −39.1±1.5 mV (N = 10). To compensate for this change in membrane potential, gluconate sweeps were analyzed using a test pulse that was 10 mV more positive than the test pulse used for analysis in control conditions. However, a large reduction in the number of channel openings was observed with gluconate at all three test potentials (data not shown). The reduction in open probability could be reversed by washout of the gluconate solution (Fig. 3). Consistent with results of variance/mean analysis shown in Fig. 2, the amplitude of channel openings that occurred in the presence of the gluconate test solution did not differ significantly from openings measured in control conditions (Fig. 3B). The finding that single channel current amplitudes were unchanged by Cl^−^ replacement also provides further evidence that single channel currents were not the result of Cl^−^ channel openings. These findings confirm results of earlier studies [16], [17] showing that the inhibition of I_Ba_ by gluconate replacement is due to intracellular effects of anions that cause a change in Ca^2+^ channel open probability.

### Channel Regions Responsible for Anion Modulation

We used different subunit combinations and CaV1.2 mutations to analyze channel regions responsible for anion effects. Table 1 shows the best-fit Boltzmann parameters and current densities for each of the channel combinations. The figures illustrate effects of gluconate replacement on different channel combinations and plot the inhibition of peak current amplitude. Gluconate produced similar effects on both current amplitude and G_max_ (values provided in the figure legends).

**Figure 4:**
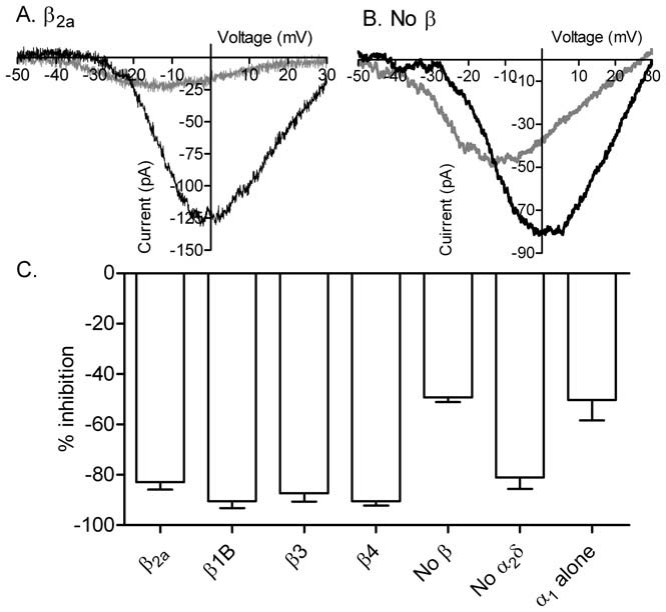
α1 and β subunits are involved in anion interactions with CaV1.2. A. I_Ba_ recorded from a cell expressing short N terminal CaV1.2 with α2δ and β2a. Currents were recorded in control conditions (black trace) and after replacing 135 mM chloride with gluconate (gray trace). I_Ba_ was recorded using a ramp voltage protocol (−90 to +60 mV, 0.5 mV/ms). B. Gluconate replacement produced less inhibition of I_Ba_ when CaV1.2 was expressed without β subunits. C. Bar graph illustrating the inhibition of I_Ba_ peak amplitude produced by gluconate replacement when CaV1.2 was expressed with β2a and α2δ (reduction in amplitude: 82.9±2.9%; N = 19; reduction in G_max_: 72.0% ), without β subunits (amplitude, −44.2±1.8%; N = 7; ΔG_max_, −46.1%), without α2δ (amplitude, −81.0±4.6%, N = 8; ΔG_max_, −80.0%), and after omission of both β2a and α2δ subunits (amplitude, −50.3±8.1%; N = 8; ΔG_max_, −49.9%). Inhibition of I_Ba_ by gluconate replacement was significantly reduced by omission of β subunits (P<0.001, unpaired t-test) or simultaneous omission of both β and α_2_δ subunits (P<0.001). Varying the β subunit composition had no significant effect (P = 0.81, ANOVA) on gluconate inhibition of CaV1.2 amplitude (β_1b_, N = 7, 90.4±2.7% inhibition of amplitude, 84.6% inhibition of G_max_; β_3_, N = 12, amplitude −87.3±3.3%, ΔG_max_ −67.8%; β_4_, N = 8, amplitude −90.4±1.8%, ΔG_max_ −76.4%).

Similar to the example in Fig. 1, Fig. 4A shows that replacement of chloride with gluconate (gray trace) caused a large decrease in the peak amplitude of the CaV1.2 current (rat CaV1.2 plus β2a and α2δ; Fig. 4A). By contrast, omission of β subunits reduced gluconate inhibition from ∼80% to −44.2±1.8% (CaV1.2/α2δ; N = 7; Figs. 4B–C). By selecting cells with large currents for the measurement of anion effects, we may have reduced differences in the amplitude of currents measured from cells with and without β subunits. Nevertheless, consistent with earlier studies [51]–[53], omission of β subunits greatly reduced CaV1.2 current density (Table 1). Omission of α_2_δ subunits also reduced current density (Table 1) but did not significantly alter gluconate inhibition of CaV1.2 currents relative to control (Figs. 4C, 4E; P = 0.51). We observed the same reduction in gluconate inhibition after simultaneously omitting both β and α_2_δ subunits as we did after omitting only β subunits (50.3±8.1% inhibition, N = 8; Fig. 4C). These results show that at least a portion of the inhibitory effects of gluconate replacement requires the presence of β subunits. However, we found no significant difference in the effects of gluconate on CaV1.2 by comparing the different β subunits β_1b_, β_2a_, β_3_, and β_4_ (P = 0.81, ANOVA, Fig. 4C).

**Figure 5:**
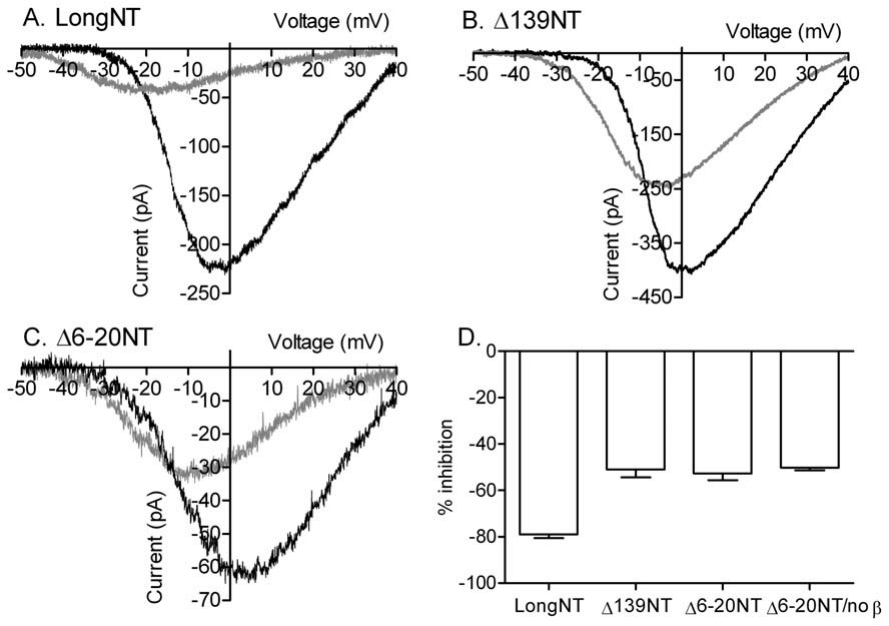
N-terminal regions involved in gluconate inhibition of CaV1.2. A. I_Ba_ from cardiac-derived long N-terminal CaV1.2 co-expressed with β2A and α2δ. The long N-terminal isoform showed a similar reduction in I_Ba_ after gluconate replacement (gray trace) as the short N terminal isoform of CaV1.2 used in previous figures. B. I_Ba_ from a mutant CaV1.2 in which the N-terminus was truncated at residue AA 139 (Δ139) recorded in control conditions (black trace) and after replacing chloride with gluconate (gray trace). C. I_Ba_ after deletion of residues AA 6–20 on the N-terminus (Δ6–20) from long N-terminal CaV1.2 in control (black trace) and low chloride (gray trace) conditions. D. Bar graph showing the percentage inhibition of I_Ba_ peak amplitude produced by gluconate replacement in the different experiments. Compared to the long N-terminal isoform of rabbit CaV1.2 (−78.2±1.5%; ΔG_max_ −71.9%, N = 16), truncating the N terminus at residue 139 (Δ139: −54.0±3.3%; ΔG_max_ −48.0%; N = 9), deletion of residues 6–20 (Δ6–20: −53.7±2.6%; ΔG_max_ −51.6%, N = 17), or expression of Δ6–20 mutant without β subunits (−50.2±1.1%; ΔG_max_ −44.7%, N = 8) all reduced inhibition of I_Ba_ by gluconate replacement from ∼75% to ∼50%.

To identify sites of anion interactions on α_1_ subunits, we used α_1_ mutants created from a long N-terminal isoform of CaV1.2 derived from rabbit cardiac cells. Similar to the rat brain-derived N-terminal CaV1.2 isoform used for earlier experiments, gluconate replacement inhibited the cardiac-derived long N terminal isoform of CaV1.2 by 78.2±1.5% (N = 16; Fig. 5A) and omitting β subunits reduced this inhibition to 50.2±1.1% (N = 8; not shown).

Although β subunits do not bind directly to the N terminus [54], β subunit enhancement of long NT CaV1.2 currents nonetheless requires a region of the α_1_ subunit N-terminus between residues AA 6–20 [33]. Expression of an α1 subunit that was truncated at AA139 to remove this region along with most of the cytosolic portion of the N-terminus (Δ139) reduced inhibition by gluconate from ∼75% to 54.0±3.3% (N = 9; Figs. 5B, D), similar to effects produced by omission of β subunits. We also tested a Δ6–20 mutant in which only this critical region was deleted from the N terminus [33]. Similar to effects produced by removal of the entire N terminus or omission of β subunits, gluconate inhibition was reduced to 53.7±2.60% (N = 17) in the Δ6–20 mutant (Figs. 5C, D). Consistent with involvement of this region in β subunit-mediated anion effects, inhibition by gluconate was not reduced any further by expressing the Δ6–20 mutant without β subunits (−50.2±1.1%, N = 8; Fig. 5D).

**Figure 6:**
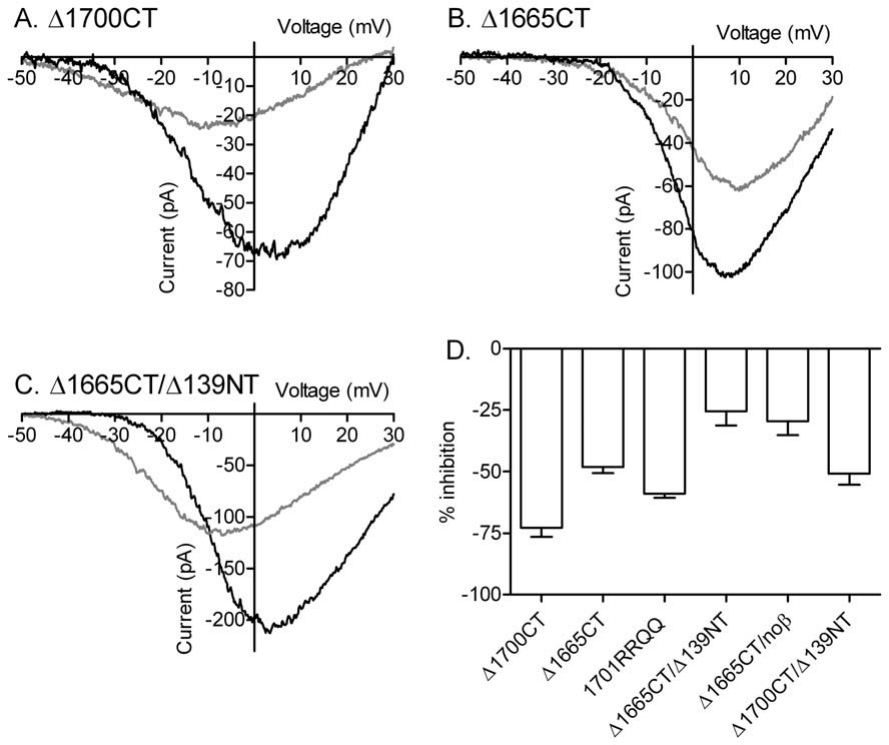
Contributions of the C terminus to anion sensitivity of CaV1.2 currents. A. Long NT CaV1.2 current after deletion of the C terminus at residue 1700 (Δ1700CT) in control conditions (black trace) and after replacing chloride with gluconate (gray trace). B. CaV1.2 current after deletion of the C terminus at residue 1665 (Δ1665CT) in control (black trace) and gluconate (gray trace) conditions. C. I_Ba_ from a mutant lacking both C- and N-termini (Δ1665/Δ139) in control (black trace) and gluconate (gray trace) conditions. D. Bar graph comparing the inhibition of I_Ba_ peak amplitude produced by gluconate replacement in the different mutants. Truncating the C terminus of rabbit CaV1.2 at AA 1700 (Δ1700) showed no reduction in gluconate inhibition (−72.3±2.0%, N = 4; ΔG_max_ −73.9%) compared to full length CaV1.2. However, removing an additional 35 residues by truncating the C terminus at AA 1665 (Δ1665) reduced gluconate inhibition significantly (P<0.0001) to 48.1±2.5% (N = 23; ΔG_max_ −45.8%). Two positively charged residues in this region were neutralized in a Δ1701 truncation mutant by replacing arginine with glutamine at AA1696 and 1697. Replacing these two residues reduced gluconate inhibition to 58.0±2.1% (N = 7; ΔG_max_ −43.0%). Inhibition was more strongly reduced in channels lacking both C- and N-termini (Δ1665/Δ139: −25.4±6.9%, N = 5, comparison to Δ1665 with β subunits, P<0.0011; ΔGmax −35.4%) as well as in Δ1665 channels expressed without β subunits (−29.4±5.8%, N = 7, comparison to Δ1665 with β subunits, P<0.003; ΔG_max_ −25.6%). Gluconate inhibition in the Δ139/Δ1700 double mutant (−50.8±4.4%, N = 7; ΔG_max_ −41.0%) did not differ from gluconate inhibition in the Δ139 mutant. Except where specified, α1 subunits were co-expressed with α2δ and β2A.

We then tested involvement of the C terminus in anion effects. Truncating the C terminus of rabbit CaV1.2 at AA 1700 [35] did not decrease gluconate inhibition (Figs. 6A, D) but removing only 35 additional residues by truncating the C terminus at AA 1665 [34] reduced gluconate inhibition from ∼75% to −48.1±2.5% (N = 23; Figs. 6B, D). This indicates that there is an anion interaction site on the C terminus somewhere between residues AA 1665 and 1700.

We tested a Δ1701 truncation mutant of CaV1.2 in which two neighboring arginine residues (AA 1696 and AA 1697) residing within this anion-sensitive C-terminal region were neutralized by replacement with glutamine [36]. Inhibition of I_Ba_ by gluconate replacement was reduced in this Δ1701RRQQ mutant to 58.0±2.1% (N = 7; Fig. 6D). This reduction in inhibition from ∼75% observed with the full length channel is not due to retention of a glycine residue at position 1701 since deletion of this residue in the Δ1700 mutant yielded the same anion sensitivity as the full length channel. This result suggests that the two neighboring arginines at positions 1696 and 1697 are involved in anion effects on the C terminus.

We examined the independence of N- and C-terminal anion regulatory regions by studying a double mutant in which the N terminus was truncated at AA 139 and the C terminus truncated at AA 1665. Consistent with additive effects between the two tails, inhibition by gluconate was reduced further in the Δ139/Δ1665 double mutant to 25.4±6.9% (N = 5; Figs. 6C–D). Inhibition by gluconate was reduced to a similar amount (−29.4±5.8%, N = 7) when the Δ1665 mutant was expressed without β subunits (Fig. 6D). Gluconate inhibition in the Δ139/Δ1700 double mutant (−50.8±4.4%, N = 7, Fig. 6D), which retains the anion-sensitive region on the C terminus, did not differ appreciably from gluconate inhibition observed with the Δ139 mutant.

The remaining ∼25% gluconate inhibition in the Δ139/Δ1665 double mutant suggests the possibility of at least one other anion interaction site. Consistent with the existence of an additional anion interaction site, enhancement of CaV1.2 by 14 mM perchlorate (+26.1±3.9%; N = 4, data not shown) was retained by the Δ139/Δ1665 double mutant.

## Discussion

### Comparison with I_Ca_ In Situ

Many of the anion effects that we found in expressed CaV1.2 channels have been observed with L-type Ca^2+^ channels *in situ*. Cardiac and skeletal muscle contractions, as well as I_Ca_ in skeletal muscle and pancreatic beta cells, are enhanced by low concentrations of perchlorate [13], [14], [16]. Similarly, we found that CaV1.2 currents were enhanced by low perchlorate concentrations. By contrast with low concentrations, we found that high perchlorate concentrations strongly inhibited both ventricular muscle I_Ca_ and CaV1.2 currents. Thiocyanate, which has a low charge density similar to perchlorate, did not greatly alter I_Ca_ in ventricular muscle [10] and we found that it also did not have much of an inhibitory effect on expressed CaV1.2 currents. The finding that inhibitory effects of replacing chloride with thiocyanate were much weaker than predicted from thiocyanate's position in the Hofmeister series could be explained by the presence of spatially restricted anion binding sites that accommodate one anion better than another.

### Channel Mechanisms

The inhibition of I_Ca_ in photoreceptors produced by replacement of chloride with gluconate and the enhancement of I_Ca_ in insulin-secreting beta cells produced by low perchlorate levels have both been shown to be due to changes in open channel probability mediated at intracellular sites [16], [17]. Consistent with these studies, the present results also indicate that application of gluconate test solutions reduced the open probability of single CaV1.2 channels by actions at intracellular sites. By contrast with effects on open probability, the amplitude of single CaV1.2 channel currents measured using cell-attached patch techniques or mean-variance analysis was not reduced by gluconate replacement. In photoreceptor cells, chloride imaging experiments show that application of low chloride gluconate solutions causes a reduction in intracellular Cl^−^ levels [17]. Similarly, we found that the gluconate solution depolarized HEK293 cells consistent with an efflux of Cl^−^ through endogenous Cl^−^ channels [41]. The reduction in open probability produced by superfusion with the gluconate solution is thus most likely due to intracellular effects of gluconate replacement since, in the cell-attached patch configuration, the extracellular channel surface remains continually exposed to high Cl^−^ levels. Also consistent with intracellular sites of anion modulation, truncating the intracellular N and C termini of the α1 subunit substantially reduced anion sensitivity. Thus, our results confirm earlier studies on endogenous channels [16], [17] showing that anions influence the amplitude of L-type Ca^2+^ currents anions by acting at intracellular sites to regulate channel open probability.

### N-Terminal Interactions

Replacing 135 mM extracellular chloride with gluconate inhibited CaV1.2 currents by ∼75–80%. However, omitting β subunits or deleting a short N terminal region of the α_1_ subunit involved in β subunit interactions (AA6–20) reduced gluconate inhibition to ∼50%. β subunits attach to the intracellular loop between transmembrane domains I and II of α_1_ subunits [54] but residues AA6–20 of the N terminus of the long NT isoform of CaV1.2 are critically important for β subunit enhancement of channel open probability [33]. Consistent with a critical role for this region in β subunit effects, there was no additional change in gluconate inhibition when the Δ6–20 mutant was expressed without β subunits.

**Figure 7:**
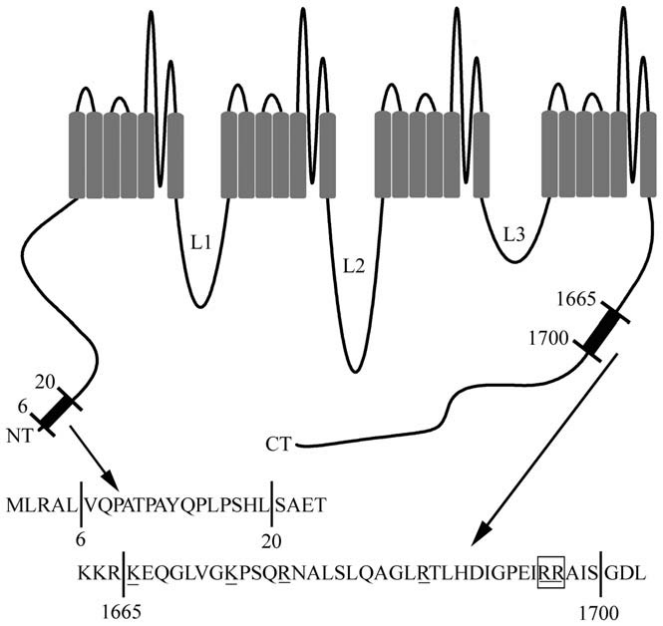
Diagram of the long NT CaV1.2 α_1_ subunit highlighting anion-sensitive regions. Our results show that anion modulation of the long NT isoform of CaV1.2 involves the interaction between β subunits and a short N terminal region between residues AA 6–20. The absence of positively charged lysine or arginine residues in this region suggests that anions do not bind directly to this region but may instead interact with residues on the β subunit. We also identified sites of anion interaction in a C terminal region between AA 1665–1700. Two neighboring arginine residues at positions 1696 and 1697 are particularly important for these interactions.

The only positively charged amino acid residues at physiological pH are lysine and arginine. Anion binding to these residues has been shown to regulate many different proteins including vertebrate opsins [19], intracellular Ca^2+^ channels [20], hemoglobin [21], [22], albumin [23], PDZ domains [24], K^+^ channels [25], [26], and kainate receptors [27]. There is also evidence that anions can influence protein function indirectly by charge shielding effects that do not require binding to specific residues [21] or by modulating the activity of serine/threonine kinases [28]–[30] and G proteins [31]. CaV1.2 and its accessory subunits possess numerous lysine and arginine residues. However, as illustrated by the diagram in Fig. 7, lysine and arginine residues are absent from AA 6–20 of the long N terminus of CaV1.2 suggesting that anions do not interact directly with this region. The shorter rat brain-derived CaV1.2 isoform used for some experiments lacks this N terminal sequence. Nonetheless, omission of β subunits caused a similar reduction in gluconate inhibition with both isoforms of CaV1.2. This further suggests that anions interact directly with β subunits rather than the N terminus. However, we found no difference in the effects of gluconate on CaV1.2 expressed with different β subunits suggesting that, if there is a direct interaction between anions and β subunits, it is likely to involve one of the more than 20 positively charged residues conserved among β_1b_, β_2a_, β_3_, and β_4_ subunits. Together, these results suggest that anions influence steric interactions between the N-terminus and β subunits that contribute to enhancement of CaV1.2 currents [33], [55], [56].

### C-Terminal Interactions

Truncating the C terminus at residue AA 1665 reduced gluconate inhibition to ∼50% but gluconate inhibition was not reduced in a mutation retaining an additional 35 residues on the C terminus. This indicates that residues within the short region between AA 1665 and AA 1700 are required for this aspect of gluconate's inhibitory effects. This region contains six positively charged residues (2 lysines and 4 arginines) that could potentially interact with anions (Fig. 7). Neutralizing two arginine residues at AA 1696 and AA 1697 by replacement with glutamine reduced gluconate inhibition from ∼75% to ∼60% suggesting that these two neighboring residues are involved in anion regulation of Ca^2+^ channel activity.

Anion effects on the C terminus were additive with anion modulation of β subunit/N terminal interactions. Truncation of either the C or N terminus alone reduced gluconate inhibition from ∼75% to ∼50%, whereas expression of a double mutant that lacked both termini reduced inhibition to ∼25%. Gluconate inhibition was also reduced to ∼25% when the C terminal truncation mutant (Δ1665) was expressed without β subunits. The residual gluconate inhibition seen in the double mutant suggests the possibility of a third anion interaction site, perhaps on the intracellular loops or more proximal tail regions of the α_1_ subunit. The possibility of a third anion-interaction site is supported by the finding that the enhancement of I_Ba_ produced by low concentrations of perchlorate persists after truncation of both N and C termini. One attractive candidate for an additional anion interaction site is a cluster of positively charged residues just proximal to residue 1665 on the C terminus.

### Functional Implications

The evidence that anions act primarily at the intracellular membrane surface has physiological implications since intracellular chloride levels vary more widely than extracellular chloride levels under both physiological and pathophysiological conditions [57]. For example, intracellular chloride levels fall by 20 mM during hyperpolarizing responses to light in retinal horizontal cells [58] and decrease by ≥15 mM during spontaneous action potential bursts in embryonic spinal cord neurons [59]. Reducing extracellular chloride by 14 mM with gluconate replacement inhibited CaV1.2 currents by ∼20% suggesting that physiologically attainable reductions in chloride levels could have potentially significant effects on I_Ca_. Consistent with a physiological role for anion modulation of L-type Ca^2+^ channels, chloride efflux through Ca^2+^ -activated chloride channels or chloride channels associated with glutamate transporters have both been shown to regulate the L-type I_Ca_ that controls neurotransmitter release from photoreceptor cells [17], [60], [61]. Similarly, chloride flux through Ca^2+^-activated chloride channels in other tissues [62] might also potentially influence I_Ca_. Chloride fluxes through volume-regulated anion channels cause swelling-induced changes in I_Ca_ that can stimulate insulin secretion from pancreatic beta cells as well as the contraction of cardiac and smooth muscle [63]–[66]. The present results suggest that, in addition to causing changes in membrane potential, swelling-induced chloride flux might exert a direct effect on I_Ca_. Effects of intracellular chloride may also help to explain the unexpected effects of chloride channel blockers on L-type I_Ca_ [10], [67].

### Summary

We found that CaV1.2 currents were influenced by the presence of various anions and could be strongly inhibited by replacement of extracellular Cl^−^ with gluconate or perchlorate. Inhibition of I_Ca_ by gluconate replacement results from actions at the intracellular membrane surface that modulate single channel open probability but not conductance. The anion sensitivity of CaV1.2 currents involves interactions between accessory β subunits and the α1 subunit N terminus along with a short region of the α1 subunit C terminus (AA 1665–1700), particularly a pair of neighboring arginine residues at positions 1696 and 1697. The evidence that anions can regulate open probability by interactions involving both N and C terminal regions of the α_1_ subunit along with the β subunit fits with the emerging view that interactions between the two tails of the calcium channel help to determine the likelihood of channel opening [35]. The strong effects of anion modulation, the sensitivity to small chloride changes, and the widespread distribution of CaV1.2 L-type Ca^2+^ channels suggest that changes in the levels of chloride and other physiological anions may be capable of influencing calcium-mediated processes in many different cell types.

## Supporting Information

Figure S1 Comparison of CaV1.2 currents measured in a cell using voltage ramps (A, 0.5 mV/ms) and steady currents during steps (B, 100 ms, 10 mV increments) in control conditions (black trace in A, circles in B) and following substitution of chloride with gluconate (gray trace in A, triangles in B).
(1.00 MB TIF)
