# Supplementary figures and images for: Anion-Sensitive Regions of L-Type CaV1.2 Calcium Channels Expressed in HEK293 Cells
Source: PLoS One. 2010 Jan 6;5(1):e8602. doi: 10.1371/journal.pone.0008602 (PMC2798859; doi:10.1371/journal.pone.0008602)

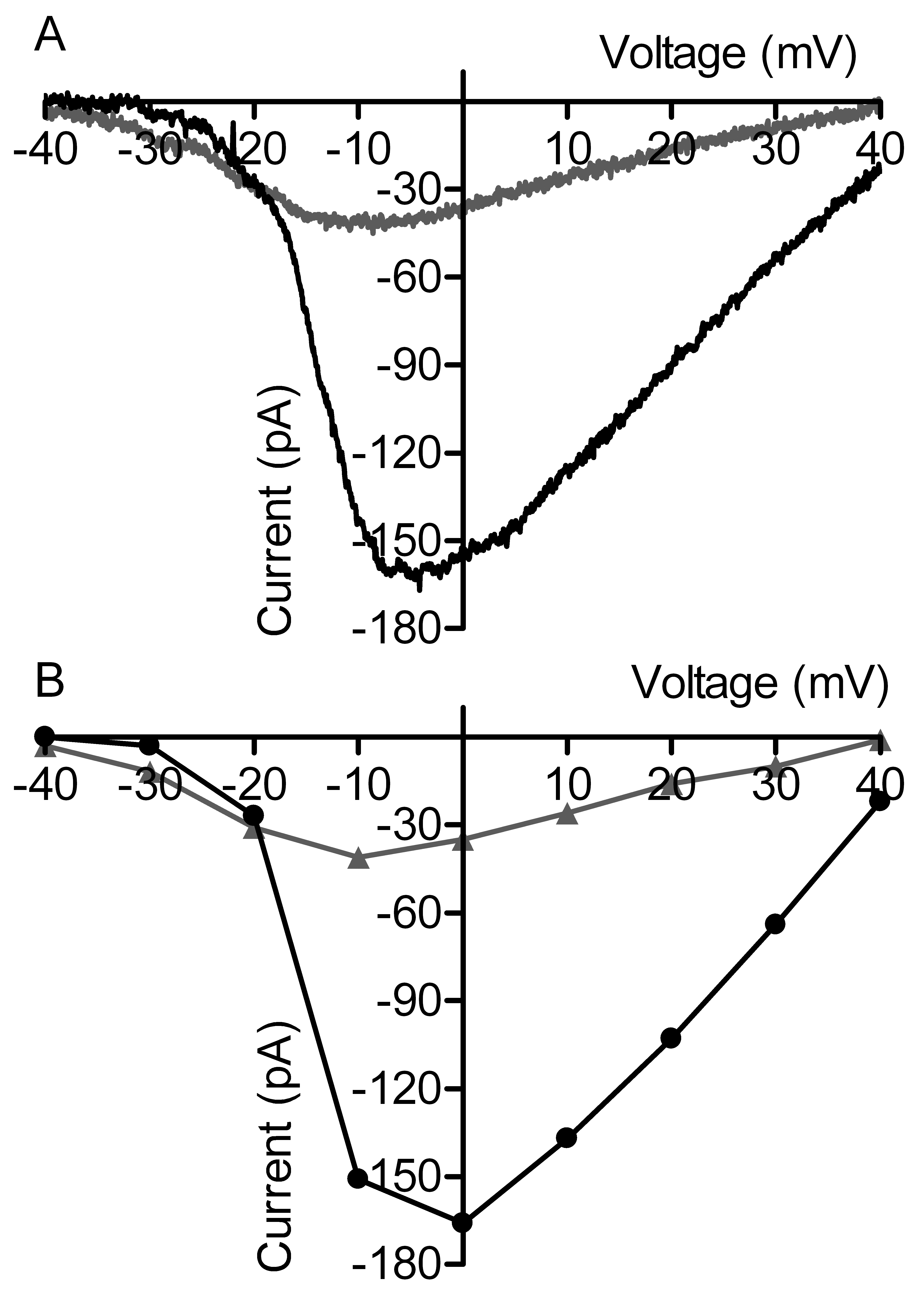

Supplement: Figure S1 — Comparison of CaV1.2 currents measured in a cell using voltage ramps (A, 0.5 mV/ms) and steady currents during steps (B, 100 ms, 10 mV increments) in control conditions (black trace in A, circles in B) and following substitution of chloride with gluconate (gray trace in A, triangles in B). (1.00 MB TIF) [file pone.0008602.s001.tif]
